# Targeting Pattern Recognition Receptors (PRR) for Vaccine Adjuvantation: From Synthetic PRR Agonists to the Potential of Defective Interfering Particles of Viruses

**DOI:** 10.3390/v9070186

**Published:** 2017-07-13

**Authors:** Andri Vasou, Nazife Sultanoglu, Stephen Goodbourn, Richard E. Randall, Leondios G. Kostrikis

**Affiliations:** 1 Department of Biological Sciences, University of Cyprus, 1 University Avenue, Aglatzia, Nicosia 2109, Cyprus; nsulta01@ucy.ac.cy; 2 Institute for Infection and Immunity, St George’s, University of London, London SW17 0RE, UK; s.goodbourn@sgul.ac.uk; 3 School of Biology, University of St Andrews, The North Haugh, St Andrews KY16 9ST, UK; rer@st-andrews.ac.uk

**Keywords:** defective interfering particles, defective viral genomes, innate immunity, vaccine adjuvants, pattern recognition receptor agonists

## Abstract

Modern vaccinology has increasingly focused on non-living vaccines, which are more stable than live-attenuated vaccines but often show limited immunogenicity. Immunostimulatory substances, known as adjuvants, are traditionally used to increase the magnitude of protective adaptive immunity in response to a pathogen-associated antigen. Recently developed adjuvants often include substances that stimulate pattern recognition receptors (PRRs), essential components of innate immunity required for the activation of antigen-presenting cells (APCs), which serve as a bridge between innate and adaptive immunity. Nearly all PRRs are potential targets for adjuvants. Given the recent success of toll-like receptor (TLR) agonists in vaccine development, molecules with similar, but additional, immunostimulatory activity, such as defective interfering particles (DIPs) of viruses, represent attractive candidates for vaccine adjuvants. This review outlines some of the recent advances in vaccine development related to the use of TLR agonists, summarizes the current knowledge regarding DIP immunogenicity, and discusses the potential applications of DIPs in vaccine adjuvantation.

## 1. Making Better Vaccines; Vaccine Adjuvants

Vaccines have proved to be one of the most successful medical interventions ever implemented; some of the greatest success stories in public health are attributed to vaccination, such as the worldwide eradication of smallpox and the near-elimination of poliovirus. Modern vaccines act by inducing a protective adaptive immune response to a pathogen-associated antigen by mimicking the naturally occurring immune response to a disease-causing pathogen but without causing disease. The initiation of innate immunity and the activation of specialized antigen-presenting cells (APCs) pave the way to a pathogen-specific long-lasting adaptive immune response. Traditionally, vaccines have comprised either live-attenuated variants of the targeted pathogen or non-living antigens, ranging from inactivated/killed pathogens to recombinant antigens [1]. Live-attenuated vaccines have good immunogenicity and are safe for most recipients; however, these types of vaccines can cause disease when administered to individuals with an unrecognized immunodeficiency and they also exhibit a potential of reversion to virulence [2]. Non-living antigen vaccines are safer for immunocompromised individuals but are often poorly immunogenic. Immunostimulatory substances, known as adjuvants, help increase vaccine immunogenicity and have been used in human vaccines for more than 80 years. Aluminum salts were the first adjuvant used in human vaccines in 1932 [3,4], and novel adjuvants have been introduced in vaccine formulations only in the last two decades [5,6]. The improvements in vaccine immunogenicity when an antigen is co-administered with an adjuvant are exemplified by the case of influenza A virus subtype H5N1 pandemic vaccines [7]. Compared to non-adjuvanted and alum-adjuvanted vaccines, oil-in-water emulsions (MF-59) have conferred significant adjuvant effects on inactivated H5N1 pandemic influenza vaccines in humans, inducing improved immunogenicity in all age ranges and cross-reactive immune protection against H5 subtype clades as well as sparing antigen, thereby allowing an effective increase in supply [5]. The H5N1 experience illustrates that vaccine immunogenicity can be remarkably improved when vaccines are administered with the appropriate adjuvant. Despite great advances in vaccine efficacy and implementation over the past several decades, infectious diseases remain the most important cause of childhood mortality [8], while respiratory infections, diarrhea and tuberculosis all rank in the top ten leading causes of death across all age groups [9]. The most important challenges in vaccine development are linked to (i) complex pathogens, such as those that cause immune dysfunction in the host (e.g., human immunodeficiency virus; HIV), those with complex life cycles (e.g., malaria) or those with a latent disease phase (e.g., *Mycobacterium tuberculosis*), and (ii) high-risk populations, such as infants (immature immunity), the elderly (immunosenescence), and chronically diseased or immunocompromised individuals (reviewed by DiPasquale et al. [10]). Recent advances in immunology, especially a greater understanding of the link between innate and adaptive immunity, allow the development of novel adjuvants that can selectively activate immunological pathways to obtain the desired immune response against a specific pathogen in distinct target populations.

Adjuvants can augment the immune response to vaccines through a variety of mechanisms, including deposition of vaccine (antigen) and the activation of innate immunity. Early innate immunity constitutes the first line of defense against pathogen invasion. Early pathogen recognition plays a crucial role in the subsequent triggering of a proinflammatory response to the invading pathogen while orchestrating pathogen-specific adaptive immune responses. Adjuvants can stimulate innate immunity by interacting with cellular pattern recognition receptors (PRRs), which detect pathogen-associated molecular patterns (PAMPs), distinct, evolutionarily conserved structures on pathogens [11]. Currently, several PRRs have been identified, including the well-characterized toll-like receptors (TLRs), retinoic acid-inducible gene I (RIG-I)-like receptors (RLRs), nucleotide-binding oligomerization domain NOD-like receptors (NLRs), C-type lectin receptors (CLRs) and the recently described cytosolic DNA sensors (CDSs) [12]. APCs, such as dendritic cells (DCs), express a repertoire of PRRs, allowing the recognition of a range of pathogenic constituents. Upon PAMP engagement, PRRs trigger complex signal cascades that lead to the production of an appropriate set of cytokines and chemokines, including interferons (IFNs), the enhancement of antigen presentation capacity and the migration of DCs to lymphoid tissues, where the DCs interact with T cells and B lymphocytes to initiate and shape the adaptive immune response. The matured DCs are also endowed with the ability to stimulate naive CD4+ T cells into different T helper (T_h_) subsets (e.g., T_h_1 and T_h_2 cells), which provide help to B cells to facilitate antibody production [13]. The differentiation of T_h_ cells is regulated by several cytokines; for example, the development of naive CD4+ T lymphocytes into T_h_1 cells is regulated by a number of cytokines including IL-12, IL-15 and IL-27 [14]. In brief, a T_h_1 response primarily develops following infection with intracellular pathogens, such as viruses and some bacteria, whereas T_h_2 cells predominate in response to large extracellular parasites [15]. Since most licensed adjuvants induce a T_h_2-type response rather than a T_h_1-type response [16], a current challenge is to develop adjuvants that induce a strong T_h_1 bias to increase the efficacy of vaccination against intracellular pathogens, such as HIV and malaria.

PRR agonists have been in the spotlight recently because of their profound immunostimulatory effects, which are associated with the induction of innate immunity. The nature of innate immunity is coupled with subsequent adaptive immunity; consequently, activators of PRRs, such as TLR agonists and poly(I:C) (reviewed below), can enhance or even tailor the immunogenicity of a given vaccine and are, therefore, considered promising molecules for developing new vaccine adjuvants. Furthermore, PRR agonists may be utilized as alternative forms of prophylactic or therapeutic agents to combat infectious diseases [13,17]. Defective interfering particles (DIPs) are mutant virus particles that contain defective virus genomes (DVGs), a subset of which are powerful activators of innate immunity Indeed, DIPs of negative-sense RNA viruses are critical danger signals for viral infection, because these particles specifically stimulate RLR signaling and, therefore, their presence instigates powerful antiviral immunity. The evident immunostimulatory activity of DIPs led to the study of defective viral particles as narrow- or broad-spectrum antivirals (reviewed by Dimmock et al. [18]) and also as vaccine adjuvants. In this review, we discuss the importance of innate immunity in acquiring pathogen-specific adaptive immunity, how PRR agonists are being developed as vaccine adjuvants, and how virus DIPs and DVGs offer advantages for the enhancement of immune responses.

## 2. Pattern Recognition Receptor Agonists: A Diverse Class of Vaccine Adjuvants

**Figure 1:**
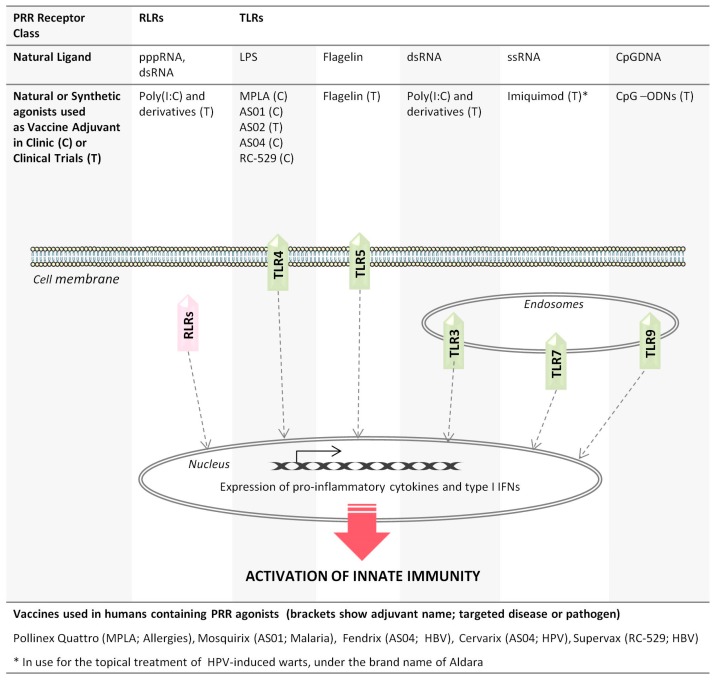
Pattern recognition receptor (PRR) agonists used as vaccine adjuvants in the clinic or in clinical trials (not an exhaustive list) referenced in the manuscript. For simplicity, figure shows TLR4 only on cell membrane, however TLR4 can signal both at cell membrane and endosomes. Abbreviations: PRR, Pattern recognition receptor; RLRs, RIG-I-like receptors; TLRs, Toll-like receptors; pppRNA, triphosphate-RNA; dsRNA, double-stranded RNA; LPS, lipopolysaccharide; ssRNA, single-stranded RNA; CpG-ODNs, CpG-containing oligonucleotides; poly(I:C), polyinosinic:polycytidylic acid; MPLA, monophosphoryl lipid A; AS, adjuvant system; HBV, hepatitis B virus; HPV, human papillomavirus.

Recent advances in the study of innate immune receptors and their ligands has laid the foundation for the development of a series of novel immunoenhancers, a number of which are currently approved for human use (Figure 1). Given that TLRs are the most extensively characterized class of PRRs, it is not surprising that most adjuvants in clinical use target TLRs (comprehensively reviewed by others [19,20,21,22]). Ten TLRs have been identified in humans and are categorized into two groups: those located at the cell membrane and the intracellular TLRs, which are expressed on the membrane of endocytic vesicles or other intracellular organelles [23,24]. TLR4 is unique among TLRs as it initiates pathways in different cellular locations including the cell membrane and intracellular compartments. The location of TLRs is directly associated with the type of microbial PAMPs they recognize. For instance, TLRs expressed on the cell membrane sense microbial membrane components, including lipids and flagella, whereas TLRs expressed in intracellular vesicles sense microbial nucleic acids, including double-stranded RNA (dsRNA), single-stranded RNA (ssRNA) and CpG DNA motifs [25] (Figure 1).

TLR-based adjuvants mimic PAMP(s) generated during a natural infection and, therefore, can be highly effective against pathogens or diseases that naturally activate the associated PRRs. For instance, TLRs play a vital role in the control of hepatitis B virus (HBV) infections in vivo, specifically by activating antiviral innate immune responses and modulating HBV-specific adaptive immunity, which is crucial for terminating the virus infection [26]. Natural or synthetic ligands of several TLRs are present in licensed human vaccines, or are currently being tested in clinical trials, as adjuvants in various vaccine formulations. These are ligands of either surface TLRs (e.g., TLR4 and TLR5) or ligands of endosomal TLRs (e.g., TLR7/8 and TLR9) (Figure 1). The adjuvant system 04 (AS04) represents one of the most successful adjuvant systems currently present in two registered vaccines: Fendrix, the HBV vaccine [5], and Cervarix, the human papillomavirus (HPV-16/18) cervical cancer vaccine [27]. AS04 combines aluminum salts and the TLR4-agonist 3-O-deacylated-4′-monophosphoryl lipid A (MPLA), a detoxified derivative of lipopolysaccharide (LPS) with retained immunostimulatory capacity [28]. More specifically, MPLA stimulates a polarized T_h_1 cell response, in contrast to the mixed T_h_1-T_h_2 cell response of aluminum salts alone [29,30], and induces considerably fewer pro-inflammatory cytokines than the parent LPS molecule [31]. In addition to AS04, the AS01 and AS02 adjuvant systems also consist of MPLA but in combination with *Quillaja saponaria* Molina fraction 21 (saponin QS-21) and a liposomal suspension (AS01) or an oil-in-water emulsion (AS02) [28]. AS01 is present in Mosquirix, the first malaria vaccine to be approved for immunization against *Plasmodium falciparum* [32]. Although AS02 was the first adjuvant to be tested in trials as an adjuvant for the malaria vaccine, AS01 induced better antigen-specific immunity to the *P. falciparum* circumsporozoite (CS) and was therefore selected for use in Mosquirix [33,34]. Several clinical trials are presently investigating the adjuvant activity of AS01 and AS02 in vaccines against HIV, tuberculosis, HBV and malaria. In addition to these MPLA-based adjuvant systems, MPLA has also been approved for use in an allergy vaccine, namely, Pollinex Quattro. Specifically, MPLA triggers a T_h_1-type immune response characterized by an increase in allergen-specific antibody levels when administered to patients suffering from seasonal allergic rhinitis [35]. Pollinex Quattro is in clinical use against seasonal allergic rhinitis in some countries, and ongoing clinical trials are also evaluating MPLA as a potential adjuvant for vaccines targeting other pathogens, including leishmania parasites and herpes virus [20]. In addition, aminoalkyl glucosaminide 4-phosphates (AGPs) represent a new class of synthetic lipid A analogs that can be manufactured at high purity as single chemical units, unlike MPLA [36]. RC-529 (also known as Ribi.529) belongs to the AGP family and is a fully synthetic monosaccharide mimetic of MPLA. Notably, RC-529 increased the immunogenicity of the human HBV recombinant vaccine Supervax, compared with that of the aluminum-adjuvanted version of the vaccine [37]. Supervax has an acceptable safety profile and is approved for vaccination against HBV in Argentina [37].

Several other TLR ligands have shown promising adjuvant activity in clinical trials (Figure 1). Imiquimod (R837) belongs to the imidazoquinoline family and is a small synthetic compound recognized by the TLR7 receptor in endosomes. Imiquimod has been successfully used to treat HPV-induced genital warts and certain skin cancers under the brand name of Aldara [38]. The use of imiquimod as a vaccine adjuvant is still under investigation; however, a recent clinical trial has demonstrated that pretreatment with topical imiquimod significantly enhances the immunogenicity of the intradermal trivalent influenza vaccine [39]. Likewise, synthetic oligonucleotides (ODNs) harboring CpG motifs (CpG-ODNs) elicit potent immunostimulatory responses through TLR9 and have shown promising adjuvant activity in both experimental and clinical settings. The immune effects of CpG-ODNs result from the activation of TLR9s expressed on DCs and B cells, which subsequently stimulate several aspects of innate and adaptive immunity, including the production of IFNs and pro-inflammatory cytokines (IL-6, TNF-α), activation of natural killer (NK) cells, and differentiation of T_h_1 immune cells [40]. CpG-ODNs have improved the immunogenicity of a commercially available HBV vaccine (Engerix-B) [41], increased the antigen-specific immune responses against anthrax [42], and demonstrated promising activity as an immunotherapy for the treatment of cancer [43]. Numerous ongoing clinical trials are investigating the therapeutic potential of CpG-ODNs as adjuvants for vaccines targeting cancer, infectious diseases and allergies [20]. Lastly, flagellin, the main constituent of bacterial flagella, is potently recognized by cell surface TLR5 and has shown promising immunoenhancing activity in novel formulations of influenza vaccines. Specifically, recombinant influenza vaccines comprising flagellin fused to influenza antigens [e.g., matrix protein 2 (M2; VAX102) or hemagglutinin (HA; VAX128)] resulted in high antibody titers, seroconversion and protection [44,45]. Flagellin-adjuvanted recombinant influenza vaccines therefore represent a promising next-generation vaccine technology.

Several synthetic dsRNAs have also been designed to mimic the natural dsRNA ligands of PRRs, such as RLRs and TLR3 (Figure 1). Among them, polyinosinic:polycytidylic acid (polyI:C) is a potent activator of the type I IFN response [46], representing a promising immunostimulatory candidate for vaccines. Poly(I:C) signaling is primarily dependent on TLR3 and MDA-5 and strongly drives cell-mediated immunity and the production of type I IFNs [47,48]. Although polyI:C is highly effective in modulating innate immunity, it was demonstrated early on that human serum has a relatively high level of enzymatic activity that causes poly(I:C) hydrolysis and inactivation [49]. Based on this phenomenon, poly-ICLC, a derivative of polyI:C stabilized with poly-l-lysine and carboxymethylcellulose, has improved pharmacokinetic properties while maintaining the immunostimulatory activity of the parental molecule [50]. Poly(I:C)/poly-ICLC elicits strong T_h_1 immune responses in mice and nonhuman primates [51,52]. Notably, type I IFN signaling through the IFN receptor (IFNAR) is required for poly(I:C) to establish T_h_1 responses to a DC-targeted HIV gag protein vaccine in mice [51,53]. Because type I IFNs have been linked to the activation of T_h_1 responses while serving as counter-regulators of T_h_2 differentiation (reviewed by Huber et al. [14]), it is believed that the ability of synthetic dsRNAs to induce T_h_1 immunity is related to their well-documented ability to induce IFNs. The effectiveness of polyI:C/poly-ICLC as an HIV vaccine adjuvant is still under investigation; numerous clinical studies are also investigating the efficacy and tolerability of poly-ICLC as an anti-retroviral agent.

Early innate immunity plays a significant role in controlling tumor progression; for this reason, PRR agonists have also been actively pursued for their anti-tumor properties and therapeutic potential as adjuvants for cancer vaccines. Current evidence suggests that type I IFN signaling participates in innate recognition of tumors and subsequently leads to a functional tumor-associated antigen (TAA)-specific T cell immunity [54,55]. In fact, spontaneous anti-tumor immunity is likely to be related to damage-associated molecular patterns (DAMPs), which are molecules that are usually released by dying or dead cells as a signal of danger. Such cancer-derived DAMPs can be recognized by PRR receptors on innate immune cells, which subsequently trigger innate immunity [56]. Therefore, the idea of stimulating PRR receptors to potentiate anti-tumor immunity has been eagerly embraced by tumor immunologists, and poly(I:C)/poly-ICLC is currently considered one of the most promising immunotherapeutic agents for improving cancer immunotherapy outcomes. The addition of poly(I:C)/poly-ICLC as a single adjuvant to different cancer vaccine formulations enhances the induction of TAA-specific T cell immunity to several tumor types, such as lymphomas, melanomas and lung cancer tumors, demonstrating promising adjuvant activity for immunotherapies [57]. The anti-tumor activity of poly(I:C)/poly-ICLC is being tested in ongoing clinical trials [58,59,60] and has been shown to be safe in humans [61]. In addition to poly(I:C)/poly-ICLC, a novel RNA-based PRR agonist (RNAdjuvant^®^) has also proven to have potent immunostimulatory effects for cancer vaccines [62] and will be employed in the therapeutic cancer vaccine formulation developed by the HEPAVAC Consortium to specifically target liver cancer [63]. Taken together, the results from clinical studies substantiate the ability of synthetic PRR agonists to initiate anti-tumor immune responses in combination with cancer vaccines, increasing their potential application in future therapeutic interventions.

Despite the evident immunostimulatory activity of PRR agonists, the use of such molecules as vaccine adjuvants still has several limitations. The cost of manufacturing, especially for synthetic agonists such as synthetic dsRNAs, remains a major limitation for their future clinical application. Expensive adjuvants increase vaccine pricing, which can limit vaccine’s worldwide distribution. Moreover, for intracellular PRR agonists, efficient delivery to target cells is vital for maximal adjuvant activity, as inefficient internalization would diminish their ability to activate PRR receptors. In currently used adjuvant systems, this issue is addressed by combining intracellular PRR agonists with carrier systems (such as liposomes and nanocarriers) [19]. This approach appears to improve the effect of the ligands by facilitating their internalization and thus potentiating their activity. Furthermore, since most of current PRR agonists target TLRs, the immune effects of these molecules are essentially restricted to immune cells, where TLRs are ubiquitously expressed. Regardless, it has been unambiguously illustrated that PRR agonists are reliable microbial mimics that efficiently stimulate innate immunity and consequently remain a promising class of new adjuvant candidates that is being further explored.

## 3. The Immunostimulatory Activity of Defective Interfering Particles of Negative-Sense RNA Viruses

Given the diversity of PRRs and the large number of their possible ligands, only a small portion of PRR ligands has been investigated as vaccine adjuvants. Therefore, identifying and understanding the mode of action of natural PRR agonists, represents a fertile area of research to broaden the molecular diversity within this class of adjuvants. DIPs of negative-sense RNA viruses are strong activators of innate immunity and could also represent attractive vaccine adjuvants that, as will be discussed, may have additional benefits over TLR agonists.

It is believed that DIPs arise spontaneously due to errors made by viral polymerases, however recent genomic and functional analyses support that DIPs are less likely to be generated randomly. DIPs contain DVGs, in which at least one gene is deleted, either entirely or sufficiently to cause a loss of function. The resulting DI viruses are defective for replication because these viruses have lost an essential gene(s) required for replication and, therefore, only replicate in the presence of a coinfecting wild-type (“helper”) virus that provides the missing functions [64,65]. DIPs are referred to as “interfering” because they attenuate the replication of the wild-type virus [66]. Owing to their smaller size, DVGs have a competitive advantage in replication rate and thus can be synthesized more rapidly by the viral polymerase; after multiple rounds of replication, the copy number of DVGs outpaces that of the wild-type virus (reviewed by Marriott et al. [67]). The ability of DIPs to interfere with wild-type virus replication was first described for the influenza virus in the 1940s [68]. The generation of DIPs has been more extensively studied in RNA viruses since the RNA-dependent RNA polymerase of these viruses lacks proofreading capacity and is therefore more prone to making errors during the replication process. However, DIPs are not an exclusive feature of RNA viruses because potentially all viruses are capable of spontaneously making mistakes during their replication cycle. DVGs have been isolated from several distinct viral families, including *Rhabdoviridae*, *Togaviridae*, *Flaviviridae*, *Paramyxoviridae*, *Papillomaviridae*, *Adenoviridae*, *Herpesviridae*, *Tombusviridae*, bacteriophages and many more (reviewed by others [18,69]). Although the accumulation of DVGs was demonstrated early on in vitro, initial investigations failed to detect DVGs in natural infections, suggesting that DVGs are laboratory artifacts. Advances in molecular techniques, especially deep sequencing analysis, helped overcome technical difficulties in discriminating between wild-type and defective genomes, leading to the identification of defective genomes in a number of human infections. DVGs were first identified from patients with viral hepatitis infections [70,71,72] and were more recently isolated from patients infected with dengue [73], influenza A virus [74] and respiratory syncytial virus (RSV) [75]. The ability of defective genomes to attenuate standard virus replication, in combination with the transmissibility of the defective genomes between individuals, underpins the potential role of DVGs in driving virus-host co-evolution, and perhaps promoting virus persistence. Nonetheless, the biological role of DIPs in the context of natural infections is still under investigation.

**Figure 2:**
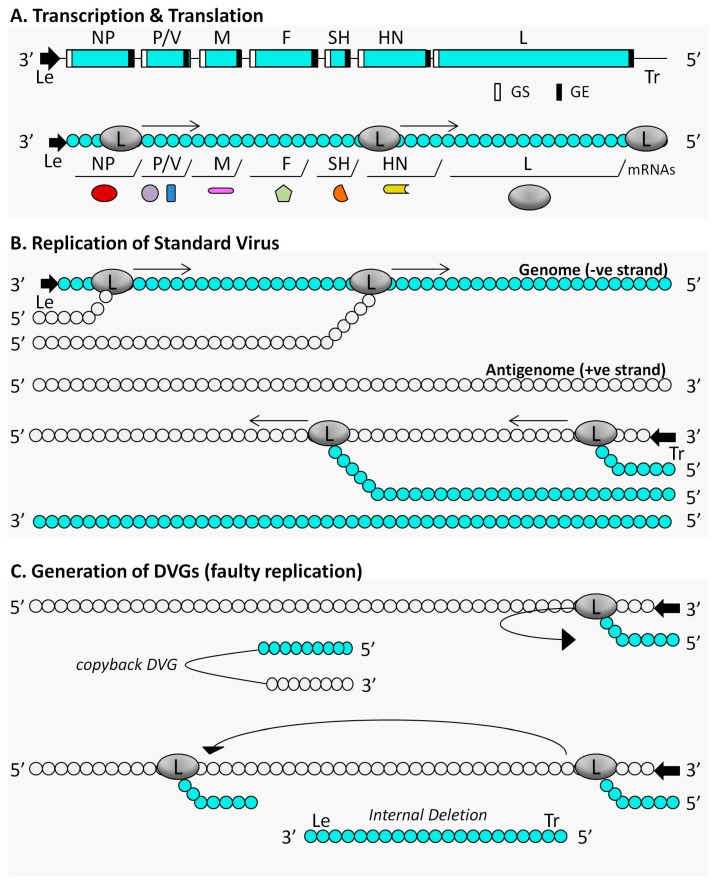
Schematic representation of defective viral genome (DVG) generation during the replication of parainfluenza virus 5 (PIV5). Genome structure of PIV5 and its mechanism of transcription (Panel A), standard replication (Panel B) and faulty replication that leads to the formation of DVGs (Panel C). PIV5 has a negative-sense single-stranded RNA genome 15,246 nucleotides long. The PIV5 genome encodes eight transcription units, and carries non-coding leader (Le) and trailer (Tr) sequences at its 3′ and 5′ ends, respectively, which are essential for controlling transcription and replication. Similar to all paramyxoviruses, PIV5 expresses an RNA-dependent RNA polymerase from the large (L) gene. The viral polymerase recognizes the genomic Le promoter and directs the synthesis of both viral mRNAs and antigenomes, which comprise the exact full-length complementary sequence of the genome. To produce separate mRNAs, the polymerase must recognize the gene start (GS) and gene end (GE) signal sequences of each gene. During replication, the full-length antigenomic RNA serves as the template for the synthesis of new genomic RNA from the antigenomic Tr promoter. At a high multiplicity of infection, the viral polymerase loses processivity, resulting in spontaneous errors. These errors are responsible for the generation of faulty genomes, known as DVGs. There are two major types of DVGs: copyback DVGs and internal deletions. Copyback DVGs maintain an authentic terminus (5′ end) and contain a segment of the viral genome flanked by a reverse complementary version of this segment. Copyback DVGs arise when the viral polymerase detaches from the template and reattaches to the nascent strand, which is then copied. The second type of DVG is generated when the viral polymerase drops off the original template and reattaches further downstream, resulting in a genomic deletion. As a result, these DVGs contain internal deletions but retain their 3′ Le and 5′ Tr sequences.

Most of the current understanding of the immunostimulatory activity of DIPs comes from studies on negative-sense RNA virus DIPs, in particular those of influenza viruses and paramyxoviruses, including Sendai virus (SeV), parainfluenza virus type 5 (PIV5) and human human respiratory virus (RSV). The immunogenicity of DIPs generated by other virus classes, such as positive sense ssRNA (+ssRNA), dsRNA viruses or different types of DNA viruses remains largely unknown, therefore this review focuses on the immune effects generated by DIPs of negative-sense RNA viruses. Two major types of DI genomes have been described for negative-sense RNA viruses: (i) copyback DVGs, which consist of a segment of the viral genome and an authentic terminus followed by an inverted repeat of this segment and the end sequence [76]; and (ii) DVGs that contain internal deletions but retain their 3′ leader (Le) and 5′ trailer (Tr) sequences and therefore can produce viral translation products [77,78]. A schematic diagram of how internal deletion and copyback DIPs are generated during the replication of the paramyxovirus PIV5 is shown in Figure 2.

**Figure 3:**
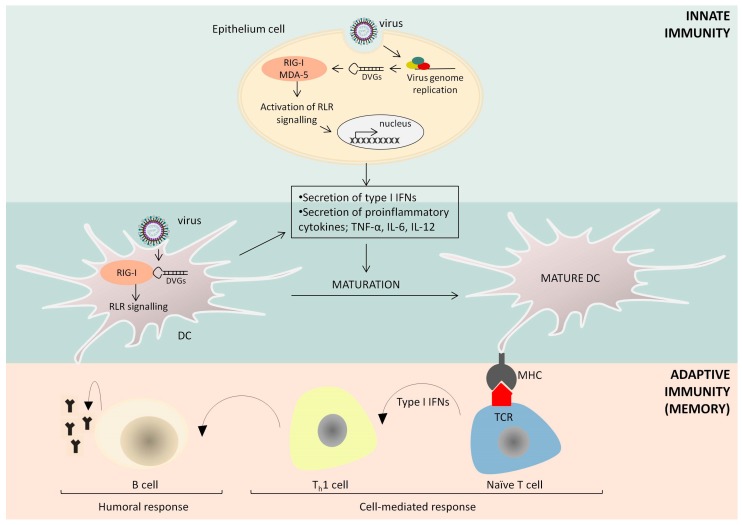
Innate and adaptive immune responses to DIPs. DIPs contain truncated forms of viral genomes, known as DVGs. Copyback DVGs have complementary ends allowing the formation of double-stranded RNA (dsRNA) structures, which can be recognized by retinoic acid-inducible gene I (RIG-I)-like receptors (RLRs), namely, RIG-I and melanoma differentiation-associated protein 5 (MDA-5). The stimulation of RLR signaling induces the expression of type I interferons (IFNs) and several proinflammatory cytokines, which all play key roles in dendritic cell (DC) maturation and the regulation of adaptive immunity. DVGs enhance the ability of DCs to activate naive T cells, increase antibody production and direct the immune response toward type 1 T helper (T_h_1) immunity, a process requiring type I IFN signaling. DIPs can initiate innate immune responses in many cell types, including epithelial cells at the site of infection and antigen-presenting cells, such as DCs. Abbreviations: TNF-α, tumor necrosis factor-alpha; IL-6, interleukin 6; IL-12, interleukin 12; MHC, major histocompatibility complex.

DIPs of negative-sense RNA viruses initiate cellular immune responses by stimulating strong signaling of intracellular RLRs, namely, RIG-I and melanoma differentiation-associated protein 5 (MDA-5), which are helicases expressed in most cell types [75,79,80,81] (Figure 3). Several studies have demonstrated that copyback genomes dominate IFN-inducing DI populations of paramyxoviruses [80,82,83,84], suggesting that unique secondary RNA structures present in these short defective genomes are perhaps driving their immunostimulatory properties. Indeed, although 5-di- or 5-triphosphates (5′-PPP) coupled to specific single- or double-stranded RNA motifs are known to trigger RLR signaling, a recent study has identified a natural viral RNA motif (SeV DVG_70–114_) that serves as a PAMP enhancer and promotes potent RLR stimulation [85]. Adding a 5′-cap structure or removing 5′-PPP significantly reduces but does not eliminate the ability of DVGs to induce IFN [83], indicating that the DVG sequence composition is also critical for effective activation of RLR signaling. Notably, although influenza viruses have not been reported to generate copyback DVGs, only internal deletions, influenza DI genomes are also capable of stimulating RIG-I signaling through a mechanism that remains to be elucidated [86].

The engagement of RLRs is strongly linked to the stimulation of innate immune responses, especially the production of type I IFNs, which elicit an antiviral function by inducing a wide array of IFN-stimulated genes (ISGs). In brief, the cellular IFN response is divided into two pathways: the IFN-induction and IFN signaling pathways. The engagement of PRRs activates a number of downstream kinases that are essential for the phosphorylation of IFN regulatory factor 3 (IRF3) and nuclear factor kappa B (NF-κB), which subsequently translocate to the nucleus to induce the IFN promoter [87]. Following its induction, IFN is secreted from infected cells and binds to the IFN receptor on the surface of infected or uninfected cells to mediate the activation of the IFN signaling pathway, which is also known as the Janus-activated kinase (JAK)/signal transducers and activators of transcription (STAT) signaling pathway [88]. More specifically, engaging the IFNAR with its ligand causes the phosphorylation of STAT1 and STAT2, which dimerize and translocate into the nucleus. In the nucleus, STATs bind to IRF9 to form interferon-stimulated gene (ISG) factor 3 (ISGF3), which is a transcription factor that regulates the expression of hundreds of ISGs. Most ISGs encode products with discrete antiviral functions, but many ISGs have still not been fully characterised [89].

Considering that DVGs of negative-sense RNA viruses are good activators of RLR signaling, it is not surprising that DIPs containing these DVGs are also potent inducers of IFNs in cell culture [90,91,92] and in vivo [75,80]. Indeed, current evidence suggests that DIPs are primarily responsible for initiating innate immune responses during paramyxovirus replication. Specifically, SeV DVGs are formed in the lungs of mice when virus replication peaks, and the presence of these genomes coincides with the induction of type I IFNs [80]. It has also been demonstrated that a recombinant PIV5 that lacks a functional V protein (termed PIV5-VΔC), which is the viral IFN antagonist, weakly activates the cellular IFN response, whereas a DIP-rich preparation of PIV5-VΔC strongly activates the induction of type I IFNs [92,93]. A recent study has reported that DVGs are the major activators of antiviral responses in human lungs during RSV infection, signifying the first evidence of an important biological role for naturally occurring DVGs during paramyxovirus infections in humans [75]. In some cases, the antiviral activity of DIPs appears to be highly dependent on the IFN system. For instance, the broad-spectrum antiviral activity of an influenza A DI virus (244 DI virus) is nearly abolished in the absence of the type I IFN system [94]. Specifically, preclinical studies have demonstrated that the ability of 244 DI virus to protect mice from non-influenza A respiratory viruses (e.g., pneumonia virus of mice and influenza B virus) requires type I IFNs as mice lacking type I IFN receptor were only poorly protected by the challenge viruses [95,96]. Although type I IFN plays a key role for the 244 DI virus-mediated antiviral activity against non-related viruses, protection from influenza A viruses does not entirely depend on type I IFNs, although type I IFNs may contribute to this protection [95,96].

The ability of DVGs of negative-strand viruses to trigger the IFN-induction cascade is not dependent on virus replication because the DVGs of several paramyxoviruses, including PIV5 and mumps virus, can induce type I IFNs in the absence of protein synthesis and consequently in the absence of infectious virus, since protein synthesis is an absolute requirement for paramyxovirus genome replication [92]. It is, however, possible that the immunostimulatory activity of DVGs requires RNA synthesis. In this regard, it is notable that it was demonstrated early on that UV-inactivated Newcastle disease virus (NDV), which had lost infectivity but retained the capacity to induce IFN, also had the ability to synthesize RNA, while exposure to larger doses of UV radiation abolished the ability of the virus to either synthesize RNA or induce IFN [97]. These early findings suggest that the virus-mediated activation of the IFN response requires RNA synthesis, perhaps because newly synthesized viral RNA serves as a template for the formation of highly immunogenic dsRNA species. Taken together, the previous studies support the notion that DVGs have an outstanding ability to stimulate an antiviral response in the presence of highly specific viral antagonists independently of type I IFNs or virus replication, highlighting that negative-sense RNA virus DIPs are critical determinants of the outcome of an infection.

DIPs not only activate the cellular IFN response but also stimulate additional aspects of host immune defense (Figure 3). For instance, DIP-rich SeV preparations can effectively induce the maturation of mouse and human DCs as measured by the up-regulation of TNF-α, IL-6 and IL-12p40 cytokines, which are indicative of DC maturation [81]. This mechanism is IFN- and TLR-independent but requires signaling through RIG-I and MDA-5, underscoring the importance of RLR signaling for DIP immunogenicity [79,81]. SeV DIPs also promote T cell activation by up-regulating the expression of cluster of differentiation 86 (CD86) and major histocompatibility complex (MHC) II molecules on the surface of DCs [79,84]. Moreover, an SeV-derived RIG-I agonist (DVG-324) enhances the ability of DCs to activate specific adaptive immune responses in vivo by stimulating the activation of IFNγ-producing CD8+ T cells and increasing antibody production [83]. As a result, immunostimulatory DI RNAs can be successfully used as tools to convert viruses with weak DC maturation abilities into potent DC stimulators [81,84]. Collectively, DIPs trigger the maturation of DCs and successfully increase antigen-specific immunity to pathogen-associated antigens.

The adjuvanticity of naturally occurring defective genomes, such as those isolated from SeV infections, has been investigated both in vitro and in vivo. Specifically, DI RNAs have exhibited promising adjuvant activity as illustrated by their ability to enhance antibody production and to also induce T_h_1 immunity when administered with inactivated vaccines or recombinant antigens [83,84,98]. Notably, a SeV-derived RNA agonist of RIG-I (IVT DI; in vitro-transcribed SeV DI) was found to induce a T_h_1-type response, enhancing the immunogenicity of an inactivated influenza A virus subtype H1N1 2009 pandemic vaccine when delivered to mice [84]. Interestingly, recombinant SeV RNAs are naked RNAs yet still immunostimulatory with an unknown route to RIG-I, an interaction which needs to be explored further. The positive results obtained from these studies indicate that natural RIG-I agonists are promising candidate adjuvant molecules that are expected to be further explored to verify their adjuvant activity in humans.

## 4. Further Applications of Defective Interfering Particles in Vaccine Adjuvantation

Even though DIPs are powerful initiators of innate immunity, synthetic dsRNAs, including sequences derived from DVGs of negative-sense RNA viruses, have received greater attention as vaccine adjuvants, perhaps because these molecules can be easily isolated as non-infectious RNA moieties. However, large amounts of DIPs have been found in currently used live-attenuated vaccines of poliovirus, measles virus and current flu vaccines [99,100,101], suggesting that the efficacy of these vaccines is related to existing DIPs. Shedding more light on the role of these naturally occurring DI RNAs in vaccine immunogenicity will evaluate their adjuvant activity and perhaps allow their further development as chemically defined vaccine adjuvants. The major challenge that arises from supplementing killed/non-replicating vaccines with DIPs is that DIPs preferably should not be contaminated with parental/infectious virus. One way to achieve this is by propagating DIPs in complementing cell lines that express the missing viral gene product(s) to support DIP formation and replication in the absence of infectious virus. In normal cells, these mutant DIPs will be deficient for replication because their defective genomes will be released in the infected cell without the ability to copy themselves and generate progeny virus particles [102]. Such recombinant DIPs would be non-infectious and would have several advantages over currently identified natural or synthetic dsRNAs. First, DIPs contain all the necessary viral components to naturally penetrate cells, which internalize the defective genomes and subsequently activate innate immunity through PRR signaling. DIPs essentially combine immunostimulatory activity and the efficiency of carrier systems. In fact, even low numbers of PIV5 copy-back DVGs were found to be capable of strongly activating innate immunity in host cell [93], denoting that DIPs are highly immunogenic. Second, DIPs combine the safety of killed vaccines and the immunogenicity of live virus vaccines and can be genetically engineered to trigger the desired immune response against a targeted pathogen. Third, DIPs are still capable of encapsidating their defective genomes to form highly stable structures. Furthermore, recombinant DIPs would have one major advantage over currently identified TLR agonists; DIPs (specifically those generated by -ssRNA viruses) are recognized by RLRs, which are expressed by almost every cell type [103]. In contrast, human TLRs are ubiquitously expressed in immune cells but less widespread in cells of non-hematopoietic origin [104]. Consequently, DIPs can be recognized as PAMPs in every cell they infect and are, therefore, more likely to potentiate high immune responses via different routes of immunization.

Although DIPs have a viral origin, their applications in vaccine development are not limited to combating viral diseases. DIPs can be used as immunostimulators in vaccines designed against other infectious pathogens (such as bacteria and parasites) and potentially diseases such as cancer. Moreover, given that all viruses, regardless of their genome type (e.g., RNA or DNA, single- or double-stranded, positive- or negative-sense), are capable of generating DIPs, it is possible that different DIPs may trigger different types of PRRs depending on DIPs’ viral origin. In this regard, it is interesting to note that PAMPs generated by DNA viruses, such as the 2′3′-cyclic guanosine monophosphate-adenosine monophosphate (cGAMP), which is produced by cyclic guanosine monophosphate adenosine monophosphate synthase (cGAS) in response to the intracellular recognition of DNA, showed great potential as an adjuvant for cutaneous vaccination in preclinical studies [105]. Briefly, cGAMP binds the stimulator of interferon genes (STING), which subsequently activates innate immune responses including the production of type I IFNs [106]. This implies that DIPs could perhaps activate different aspects of innate immunity, increasing the likelihood of activating the desired immune responses to a given pathogen. However, this is an area to be explored further. In conclusion, current evidence supports that DIPs are potent activators of innate immunity and, therefore, DIPs represent promising immunostimulatory molecules to be further investigated as a novel class of adjuvant candidates.

## 5. Conclusions

For a variety of reasons, modern vaccinology has increasingly focused on non-living vaccines that often require the addition of adjuvants to provide stimulatory signals to activate innate immune responses. However, there is no single set of characteristics that describes an ideal vaccine adjuvant for all situations. Indeed, vaccine studies using live-attenuated pathogens support the hypothesis that activating multiple innate receptors is better than activating only one receptor, indicating that adjuvant combinations may achieve a better effect. Several preclinical and clinical studies are currently investigating the efficiency of different adjuvant combinations, supporting the view that multiadjuvanted vaccines could represent the way forward for the design of new vaccine formulations. Expanding the repertoire of adjuvants enables the use of different molecular combinations to activate the desired arms of the immune system and adapt the adjuvant to a given target pathogen and/or population.

Enhancing vaccine immunogenicity by using appropriate adjuvants will also reduce the amount of immunogen required to induce protective immunity, potentially increasing the amount of vaccine that can be manufactured, having important implications for the global vaccine supply, and thereby reducing the morbidity and mortality of vaccine-preventable diseases (VPDs). In fact, the first aim of the CDC’s strategic framework for global vaccination for 2016–2020 is to control, eliminate or eradicate VPDs to reduce death and disability globally [107]. The achievement of this goal will lead to a world free of polio, the elimination of measles and rubella/congenital rubella syndrome, the control of other VPDs by vaccine introduction and the development of new vaccination strategies, including new adjuvant approaches. There is also an important need to develop vaccines with a more defined composition to improve vaccine acceptance by the public. The lack of trust in vaccines is a growing threat to the success of global vaccination programs. Vaccine hesitancy, as defined by a delay in the acceptance or the refusal of vaccines, is held responsible for reducing global immunization coverage and increasing the risk of VPD outbreaks and epidemics. In this regard, newly designed adjuvants, including potentially DIPs, with well-defined immunostimulatory activity will accelerate our efforts to develop a new generation of vaccines with a lower risk-to-benefit ratio.
